# Occurrence of Fumigants and Hazardous Off-gassing Chemicals in Shipping Containers Arriving in Sweden

**DOI:** 10.1093/annweh/wxw022

**Published:** 2017-01-01

**Authors:** Urban Svedberg, Gunnar Johanson

**Affiliations:** 1 Work Environment Toxicology, Institute of Environmental Medicine, Karolinska Institutet, IMM, Box 210, Nobels väg 13, SE-171 77 Stockholm, Sweden

**Keywords:** freight containers, occupational exposure, sea containers

## Abstract

Containerized cargo shipment makes up the backbone of international trade. The principal aim of this cross-sectional study was to establish a qualitative and quantitative description of gaseous fumigants and volatile off-gassing substances facing workers tasked with entering shipping containers. A total of 372 packed and 119 empty shipping containers were sampled in six ports and two distribution centers in Sweden. Fourier-transform infrared spectrometry (FTIR) and photoionization detection (PID) were the analytical methods applied to the bulk of samples. A small number of adsorbent samples were analyzed using gas chromatography–mass spectrometry (GC–MS). The results were compared to Swedish occupational exposure limits (OELs), the closest parallel to relevant work situations. Based on the FTIR analyses, 30 of 249 (12%) containers arrived with concentrations of fumigants and off-gassing substances above the 8-h OELs and close to 7% were above the short-term exposure limits. Eight detected chemicals were classified as carcinogens and 4% of the containers arrived with levels of carcinogens above the OELs, at a maximum 30 times the 8-h OEL. Considerable differences were observed between ports, ranging from 0 to 33% of containers arriving with concentrations above the OELs. It is believed that all observation results, apart from a single instance of a confirmed fumigant, phosphine, at 3 p.p.m., and possibly three instances of carbon dioxide, can be attributed to off-gassing substances. The FTIR methodology proved useful for quick preliminary checks and in-depth screening and identification. The PID method produced both false-negative and false-positive results where only 48% matched the FTIR observations. Adsorbent sampling with GC–MS analysis was useful for confirming volatile organic compounds but was deemed too slow for day-to-day screening. The high frequency of contaminated containers, the detection of several carcinogens, and the sporadic occurrences of high levels of fumigants are serious concerns that need to be properly recognized in order to protect the workers at risk.

## Introduction

The dawn of containerized cargo shipment in the late 1960s and early 1970s has completely changed global trade. Today, the seamless movement of intermodal containers between trucks, trains, and ships constitutes the backbone of international trade reducing dramatically the costs for shipping goods over long distances. According to the latest and most dependable and comprehensive statistics, 651 million 20-foot equivalent units (TEUs, 40-foot containers are counted as two TEUs) were handled globally in 2013 (UNCTAD, 2014). This figure, the port throughput, includes a combined measure of the number of loadings, unloadings, repositionings and transshipments of containers. Shipment in containers is likely to continue to increase in the coming years and the preliminary figure for 2014 being 684 million TEUs.

The engineering achievements invested in modern super-size container vessels and container ports, the efficiency of the logistics, and the loading and unloading times for a ship have all unquestionably contributed to a reduction in the number of historically laborious tasks, particularly in ports. However, the ‘new’ occupational hazards associated with the manual unpacking of the goods from inside shipping containers remain poorly addressed. The design of the common dry cargo container allows only limited natural ventilation during transport. Thus, any volatile chemicals added (e.g. fumigants) or emitted from the goods (off-gassing) will accumulate and may reach high levels in the container air. Unwitting workers who enter containers when they arrive may be exposed to these airborne chemicals.

The only earlier peer-reviewed publication concerning screening of shipping containers for toxic substances involved the investigation of 2113 containers in the Port of Hamburg in 2006 (Baur *et al.*, 2010). The most frequent contaminants found were formaldehyde (59% of the containers), benzene (19%), and, among the fumigants, methyl bromide (14%), phosphine (4.5%), and chloropicrin (1.7%). In 0.6% of the containers, the concentrations exceeded the levels classified as immediately dangerous to life or health (IDLH) as published by the US National Institute for Occupational Safety and Health (NIOSH, 1994). The highest level reported was 36000 p.p.m. phosphine or 120000 times the acute IDLH of 0.3 p.p.m. This concentration was also twice as high as the lower explosive limit of 17900 p.p.m. The product categories most likely to be contaminated were shoes, furniture/household goods, and foodstuffs. No detailed list of detected chemicals was reported.

There is a report in the nonpeer-reviewed literature of an investigation of 300 randomly selected import containers in the Port of Rotterdam in 2002 (Knol-de Vos, 2002). Sulfuryl fluoride, methyl bromide, phosphine, formaldehyde, ammonia, carbon monoxide, and carbon dioxide were found. In 15 (5%) of the containers, the concentrations of phosphine, methyl bromide, and formaldehyde exceeded the Dutch 8-h occupational exposure limits (OEL).

Measurements from 42888 containers in the Benelux container terminals in 2010, showed that 11% of nonfood containers and 20% of food containers had levels above the OELs (Luyts and Mück, 2011). The results were grouped according to type of cargo and the maximum concentration within each group was reported.

In a surveillance study from 2012, published by Safe Work Australia (2012), personal exposure to 13 select chemicals was recorded in 74 out of 76 investigated containers. The most frequently detected volatiles were toluene, xylenes, ethyl benzene, and methyl bromide. None of 12 personal samples, covering the entire duration of unpacking a container (2–3 h), exceeded the Australian national 8-h workplace exposure standards. However, in six containers (8%), peak exposure levels to chloropicrin and formaldehyde exceeded the short-term exposure limits (STELs).

In a pilot study, 101 randomly selected packed containers that had arrived in the Port of Gothenburg, Sweden, were screened (Svedberg and Johanson, 2011). Most containers had detectable levels of volatile chemicals, most commonly methanol (78% of the containers), hydrocarbons (47%), carbon monoxide (45%), and ammonia (15%). Carbonyl sulfide, a possible fumigant, was found in one container (1 p.p.m.). Overall, 7% of the containers had levels above or well above the Swedish 8-h OEL for at least one substance.

It may take several hours to unload a container. The exposure limit that corresponds most closely to this work situation is the 8-h OEL and the arrival concentrations found in the present study were compared to current Swedish OELs. However, this is not to imply that the measured concentrations represent the time-weighted personal exposure during the unpacking of a container. In a previous study, the time-weighted average exposure during unpacking passively ventilated (open doors) 40-foot containers ranged between 1 and 7% of the arrival concentrations (Svedberg and Johanson, 2013). Peak exposures of up to 70% of the arrival concentration were reported immediately after opening the container doors, and it may be relevant to monitor compliance with short-term exposure limits. Likewise, some occupational groups, such as customs and food inspectors, may briefly need to go deep into unventilated containers, often allowing no time for proper ventilation. Such groups will frequently be at high risk of entering an atmosphere containing harmful levels of toxic chemicals. Needless to say, odor should not be used as a means to detect containers that pose a risk or to estimate exposure levels. Many hazardous chemicals do not have warning odors and even when they do it is difficult to determine the exposure level and associated health risk from odor alone.

The current study follows up and extends an earlier pilot study carried out in the Port of Gothenburg in 2010 (Svedberg and Johanson, 2011). The principal aim was to collect qualitative and quantitative data on fumigants and off-gassing chemicals in imported containers. Reliable and interpretable data are essential for proper assessment of health risks and strategies for prevention, the consequences of changing production methods, establishing time trends, and evaluation of the impact of new regulatory requirements. Good-quality information is also needed in developing sampling strategies and selecting detection equipment.

## Methods and Instrumentation

### Study locations

Six container ports and two inland distribution centers in Sweden were selected for this cross-sectional study. The ports represent different geographical locations in an attempt to obtain unbiased samples for national assessment. The field survey was carried out in August 2013. The data from the previous pilot study in one port in May 2010 were integrated into the results (Svedberg and Johanson, 2011). The ambient temperatures during both field campaigns were in the range of 20–25°C.

### Mobile laboratory

A motor home was acquired to serve as a temporary mobile laboratory and office. A bench-top Fourier-transform infrared (FTIR) spectrometer was installed together with an extraction sample pump and 40-m tubing routed to the target containers. The motor home was repeatedly repositioned in order to reach the containers. It was also used to service and calibrate other handheld sampling equipment.

### Selection of containers

In each location, the target containers were identified in consultation with the local staff. The only requirement was that import containers had not been opened after arrival and that the seals were unbroken. The containers were 20- or 40-foot dry cargo containers. This type of container is normally fitted with two or four top corner vents for pressure equalization. Loaded import containers and empty export containers were included. Empty containers were import containers that had been unpacked in distributions centers and returned to the port for export. They were included in the study to detect lingering residual levels as workers will enter empty containers for cleaning and during the next packing. The containers were selected randomly within each category. When containers were stacked, only the two lower levels were sampled due to fall hazards. The origin, destination, and contents of the containers were initially unknown. Crude lists of the contents (or the name of the importing company) were obtained from some ports, based on the identification numbers of tested containers. Most foodstuff containers pass through dedicated import channels and were not part of the random selection process in this study. Altogether, 372 packed and 119 empty containers were sampled. This number includes 97 of the packed containers from the previous pilot study.

### Sampling

Access to the air space inside the closed containers was facilitated by the use of a specially manufactured tool, i.e. a 40-cm stainless steel tube (outer diameter 20 mm) with a flat nozzle (outer dimensions 20 × 3 × 0.5 cm) and two 9-mm entry holes near the tip. The tool was passed through the rubber lips between the container doors reaching ~10 cm inside the doors. Container air was then extracted through the tool by means of the built-in pumps in the analytical instruments.

In order to examine if sampling position affect the results, the sampler tool was inserted at the bottom, in the middle, and at the top of the door. The container air was then sampled and analyzed using a photoionization detector (PID, see description below). These measurements were performed in 31 containers with elevated volatile organic compound (VOC) levels. In the previous pilot study, all samples were collected at the bottom, while in the present study, by default, samples were collected in the middle and at the top only.

### Analytical methods

#### Photoionization detection

A collective non-specific measure of the VOCs was obtained by using a handheld PID (pppRAE Plus, RAE Systems, San Jose, CA, USA) equipped with a 10.6-eV lamp. It was calibrated against toluene, and the results were expressed as toluene equivalents. As the Swedish OEL for toluene is 50 p.p.m., an OEL of 20 p.p.m. was used when evaluating the PID results in order to provide some latitude for the non-specificity of the PID measurements. A steady reading was normally obtained within 1 min. The lowest PID readout was 1 p.p.b. Background (outdoor air) levels ranged between 0 and 100 p.p.b. (typically 50 p.p.b.). Hence, 100 p.p.b. was considered a practical detection limit. In total, 260 packed and 119 empty containers were analyzed using the PID instrument. The PID was not used in the previous pilot study in Port 6. The PID method was used as the primary method for randomized sampling and the descriptive presentation of variation between locations.

#### Fourier-transform infrared spectrometry

Volatile substances including VOCs and inorganic gases were analyzed using a FTIR instrument (MB 3000, Bomem Inc., Quebec, Canada) with 1 cm^−1^ spectral resolution and a deuterated triglycine sulfate detector. The instrument was fitted with a 2-l analytical gas cell with a fixed 10-m optical path length and KBr windows (Gemini Scientific Instruments, Buena Park, CA, USA). Qualitative and quantitative analyses of gases and volatile compounds were based on library spectra from Infrared Analysis Inc. (Irvine, CA, USA). A diaphragm pump (KNF Type NO26 1.2 AN.18, KNF Neuberger GmbH, Freiburg-Munzingen, Germany) sampled container air through a sample line with a 6-mm inner diameter routed from the containers to the FTIR, delivering an effective flow rate of 5 l min^−1^.

The FTIR spectra were collected statically (i.e. with the sampling pump turned off), typically by averaging 16–32 spectra. An initial spectral quality check was made on-site, but final analyses were performed in the home office. After subtraction of positively identified substances, a residual IR-signal remained in ~75% of the samples. The residual signal represented a mixture of several chemicals that could not be identified. They typically had a general resemblance to various aliphatic hydrocarbons, particularly the alkanes, often with a close, but not perfect, match for *n*-octane. The residual signal was therefore reported as octane equivalents based on the signal response in the C-H-stretch region (i.e. 3050–2800 cm^−1^). Hexanes (except *n*-hexane), heptanes, and octanes have Swedish 8-h OELs of 200 p.p.m., whereas pentanes, *n*-hexane, and nonanes have OELs of 600, 25, and 150 p.p.m., respectively (SWEA, 2015). For the purposes of this report, the octane residual was compared to an OEL of 50 p.p.m. This value is close to many of the established OELs of common volatiles organic compounds (VOCs) and provides some latitude for the unknown characteristics of the residual. In total, 249 packed containers were analyzed using the FTIR method. The containers selected did not fully match those analyzed using the PID method. Because of the longer time required for sampling and analysis, a smaller number of containers was analyzed in those locations where many containers were available. In the previous pilot study, only FTIR analyses were made.

The minimum detection levels (MDLs) for all identified compounds are listed in Table 2. The MDLs are calculated as twice the peak-to-peak noise in an absorbance spectrum generated from two consecutive single-beam spectra collected with the gas cell filled with clean air, i.e., with no gaseous compounds present except naturally occurring background levels of water and carbon dioxide. In practice, the actual MDLs in a spectrum obtained from a container air sample may be higher, due to overlapping spectra from other compounds. The MDLs are, whenever appropriate, based on strong peaks in the finger-print region (1350–600 cm^−1^) rather than the strong bands in the C-H-stretch region, which usually suffer from severe overlapping. Furthermore, the listed MDLs are based on the actual conditions and settings selected during the field activities and do not represent technically achievable MDLs using the FTIR method.

#### Gas chromatography–mass spectrometry

Collection was on adsorbent tubes in 21 cases where the initial PID or FTIR results indicated elevated levels and in three cases when the container was marked as holding dangerous goods. The main purpose was to confirm the FTIR results and to detect substances where the FTIR method has limited sensitivity or specificity. Container air was collected in Tedlar® sample bags and subsequently pumped (SKC Model 224-PCXR7) through adsorbent tubes (Anasorb 747) at a flow rate of 200 ml min^−1^. The average sample volume was ~6 l. The adsorbent tubes were extracted with dichloromethane and analyzed using GC–MS, with a phenyl-dimethylpolysiloxane column and scan mode. The VOCs were determined in the 80–300°C boiling point range and expressed as toluene equivalents. Aside from a quantitative estimate of the total VOCs and benzene, the method qualitatively identified aromatics, aliphatics, terpenes, aldehydes, ketones, alcohols, chlorinated compounds, and glycol ethers. The analytical package was not specifically designed for use on emissions in containers but targeted general emissions in indoor environments. The GC–MS analyses were carried out by an accredited (SS-EN ISO/IEC 17025) and certified (SS-EN ISO 9001) laboratory (Eurofins Pegasuslab AB, Uppsala, Sweden).

### Emissions from the container floor

In the pilot study (Svedberg and Johanson, 2011), low levels of methanol and carbon monoxide were almost always present in container air, even that in empty containers. To examine the potential contributory role of the plywood floor, a section of marine plywood flooring obtained from a container repair shop was cut into pieces measuring ~3 × 10 cm and placed in a 25-l glass flask that was then sealed. After 70 h at room temperature, a head-space sample of the atmosphere inside the flask was collected and analyzed using FTIR.

## Results

An overview of all samples reported is presented in Table 1.

**Table 1.** Overview of all samples collected in packed and empty containers by location and type of analysis.

Location	PID	FTIR	GC–MS
	Number of samples	Number of samples	Number of samples
DC1	41	35	2
DC2	23	11	1
Port 1	55	21	0
Port 2	49	21	9
Port 3	22	19	3
Port 4	20	16	1
Port 5	50	29	5
Port 6 pilot study	0	97	0
**Sum of all packed containers**	**260**	**249**	**21**
Empty return containers	119	16	0

Empty return containers were sampled at several locations.

### FTIR results

Forty-seven individual substances were identified in 249 separate containers, listed in Table 2. Eight substances were classified as carcinogens, according to the Swedish Work Environment Authority (SWEA, 2015). Thirty containers (12%) had levels equal to or above the Swedish 8-h OELs. This number includes four containers where two or more substances were found with similar toxicological properties, each below their respective OEL, but when combined were above unity. In 17 (6.8%) of the containers, the levels were equal or above a statutory 5-min ceiling limit or a recommended 15-min STEL.

**Table 2.** Substances detected in 249 randomly selected packed import containers using FTIR gas phase analysis.

Chemical	CAS number	Containers identified	Containers identified	Median concentration	Maximum concentration	OEL^a^	Containers exceeding OEL	Maximum/OEL	MDL
		Number	%	p.p.m.	p.p.m.	p.p.m.	%	Ratio	p.p.m.
Acetaldehyde	75-07-0	1	0.4	17	17	25	0	0.7	0.96
Acetone	67-64-1	15	6	2	97	250	0	0.4	0.32
Ammonia	7664-41-7	40	16	0.2	72	20	0.8	4	0.09
**Benzene**	71-43-2	1	0.4	8.6	8.6	0.5	0.4	17	0.14
Butanone, 2- (MEK)	78-93-3	1	0.4	27	27	50	0	0.5	0.5
Butyl acetate	123-86-4	1	0.4	0.3	0.3	100	0	0.003	0.04
Carbon dioxide	124-38-9	14	6	193	8300	5000	1.2	1.7	^b^
Carbon monoxide	630-08-0	191	77	1.5	43	35	0.8	1.2	0.14
Carbonyl sulfide	463-58-1	4	1.6	0.6	1.5	5^c^	0	0.4	0.01
**Chloroethanol, 2**-	107-07-3	1	0.4	6.3	6.3	1^d^	0.4	6	0.34
**Chloroform**	67-66-3	1	0.4	1	1	2	0	0.5	0.05
Cyclohexane	110-82-7	5	2	3.2	40	200	0	0.2	0.02
Dichloro-1-fluoroethane, 1,1- (HCFC-141b)	1717-00-6	3	1.2	0.3	0.3	N/A	0	N/A	0.13
**Dichloroethane, 1,2**-	107-06-2	2	0.8	20	30	1	0.8	30	0.31
**Dichloromethane**	75-09-2	4	1.6	2.6	22	35	0	0.6	0.22
Dimethoxymethane	109-87-5	5	2	1.2	5.2	1000^c^	0	0.005	0.12
Dimethyl ether	115-10-6	1	0.4	9.6	9.6	500	0	0.02	0.25
Ethanol	64-17-5	17	7	4.3	92	500	0	0.2	0.49
Ethyl acetate	141-78-6	12	5	0.8	10	150	0	0.07	0.06
**Ethylene oxide**	75-21-8	1	0.4	1.7	1.7	1	0.4	1.7	0.22
Ethylene	74-85-1	2	0.8	0.8	1	250	0	0	0.14
**Formaldehyde**	50-00-0	9	4	0.6	2	0.3	2.8	7	0.08
Heptane, *n*-	142-82-5	1	0.4	47	47	200	0	0.2	0.07
Hexyl acetate, *n*-	142-92-7	1	0.4	7	7	N/A	0	N/A	0.05
Isobutane	75-28-5	2	0.8	58	107	1000^e^	0	0.11	0.03
Isobutanol	78-83-1	2	0.8	4.6	7.9	50	0	0.2	0.28
Isobutylene	115-11-7	1	0.4	14	14	N/A	0	N/A	0.18
Isopentane	78-78-4	15	6	12	63	600	0	0.1	0.06
Isopropanol	67-63-0	1	0.4	1.7	1.7	150	0	0.01	0.8
Methane	74-82-8	8	3	6.2	14	N/A	0	N/A	0.07
Methanol	67-56-1	217	87	2.7	127	200	0	0.6	0.19
Methyl formate	107-31-3	1	0.4	1.3	1.3	100	0	0.01	0.12
Methyl methacrylate	80-62-6	1	0.4	1.5	1.5	50	0	0.03	0.1
Octamethylcyclotetrasiloxane	556-67-2	4	1.6	0.1	0.8	N/A	0	N/A	0.01
Octane equivalents (residual)		186	75	1.4	505	50^f^	0.8	10	0.08
Pentane, *n*-	109-66-0	1	0.4	62	62	600	0	0.1	0.08
Phosphine	7803-51-2	1	0.4	3	3	0.1	0.4	30	0.5
Pinene, b-	127-91-3	4	1.6	3.1	3.7	25	0	0.2	0.45
Pinene, α-	80-56-8	25	10	1.8	176	25	1.2	7	0.61
Propylbenzene, *n*-	103-65-1	1	0.4	3.4	3.4	N/A	0	N/A	0.8
Styrene	100-42-5	6	2	2.3	8	10	0	0.8	0.36
Tetrafluoroethane, 1,1,1,2- (HFC-134a)	811-97-2	1	0.4	1.9	1.9	500	0	0.004	0.07
Toluene	108-88-3	19	8	10	190	50	1.2	4	0.31
Trichloroethane, 1,1,1-	71-55-6	1	0.4	3.4	3.4	50	0	0.07	0.1
**Trichloroethylene**	79-01-6	3	1.2	0.2	0.4	10	0	0.04	0.41
Xylenes	1330-20-7	9	4	1.2	27	50	0	0.5	0.35
White spirit (<2% aromatics)		1	0.4	23	23	50	0	0.5	0.09

Chemicals listed in bold are classified as carcinogens by the Swedish Work Environment Authority (SWEA, 2015). Median values are based on all recordings above the MDL. The measured values are arrival concentrations in unopened containers and do not represent expected exposures during personal sampling. The OELs are used for comparison purposes only.

^a^OEL, Swedish occupational exposure limit (SWEA, 2015). N/A, no Swedish OEL available.

^b^MDL for carbon dioxide was set to 100 p.p.m. above that in ambient air (~380 p.p.m.). The FTIR method uses a reference spectrum of ambient air and natural CO_2_ is zeroed out.

^c^Threshold limit value (ACGIH, 2015).

^d^Swedish 5-min ceiling limit (SWEA, 2015).

^e^STEL value (ACGIH, 2015).

^f^Arbitrarily assigned OEL for this study, representing residual hydrocarbons expressed as octane equivalents.

Formaldehyde was observed in seven (2.8%) containers in concentrations above its OEL; the highest observation was 2 p.p.m., seven times the OEL. 1,2-Dichloroethane was recorded at 30 times the OEL, and benzene at 17 times the OEL, both observations were from the same container that was carrying shoes. The most serious observation from an acute toxicity viewpoint was that of 2-chloroethanol at 6.3 p.p.m., six times the 5-min ceiling limit. This particular container was also identified as having ethylene oxide at 1.7 p.p.m. (OEL 1 p.p.m.). Chloroethanol has traditionally been used as an antecedent in the manufacture of ethylene oxide. It was not known what was in this container. All these chemicals are classified as carcinogens.

None of the investigated containers had valid signs indicating that they had been treated with fumigants. The only indisputable observation of a fumigant, 3 p.p.m. phosphine (OEL 0.1 p.p.m.), was made in a container carrying rice bags. The following fumigants were not detected: chloropicrin [MDL 0.14 p.p.m., OEL 0.1 p.p.m. (TLV ACGIH, 2015)], hydrogen cyanide (MDL 0.1 p.p.m., OEL 1.8 p.p.m.), methyl bromide (MDL 2.7 p.p.m., OEL 5 p.p.m.), and sulfuryl fluoride [MDL 0.02 p.p.m., OEL 5 p.p.m. (TLV ACGIH, 2015)].

### PID results

**Figure 1.**
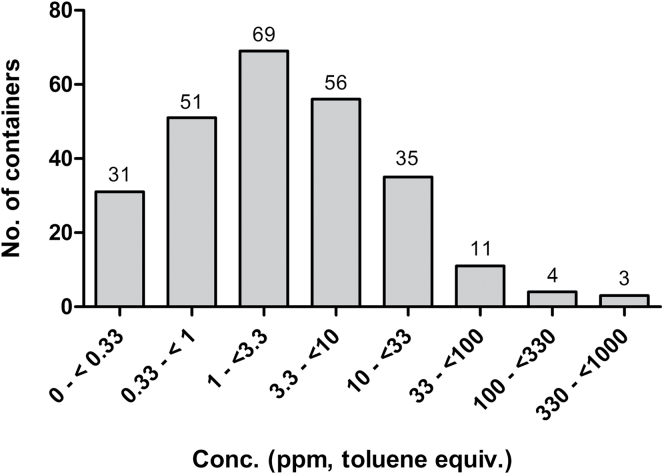
Frequency distribution of PID readouts in air sampled from packed import containers (*n* = 260).

**Figure 2.**
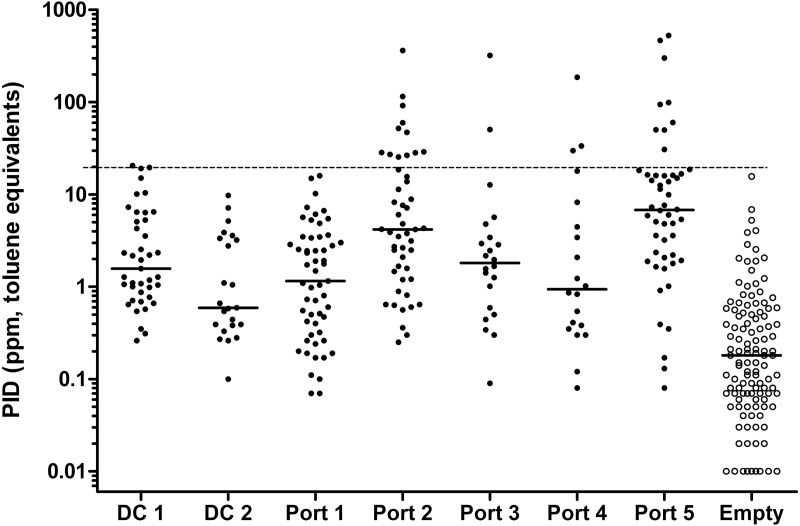
PID readouts in sealed import containers situated in two distribution centers, five container ports, and the combined samples of empty containers in several locations. The horizontal lines represent median values (–) and an arbitrarily assigned 8-h OEL (- - - -) of 20 p.p.m.

The frequency distribution of the PID readouts from 260 packed containers is shown in Fig. 1, and the spread at each location is illustrated in Fig. 2. In 27 containers (9.8%), the levels were equal to or above the subjectively assigned OEL of 20 p.p.m. (toluene equivalents) and in 51 containers (18.5%) >10 p.p.m. The median concentration in 119 empty containers was 0.2 p.p.m. compared to 2.3 p.p.m. in the packed containers. No empty container was recorded with a VOC level > 20 p.p.m. It is of note that the highest recordings in empty containers were generally obtained from fairly new units; hence, solvents from curing paint may have contributed to these higher levels. This was confirmed by the FTIR analyses, where xylenes were found in several new empty units.

### PID versus FTIR

**Figure 3.**
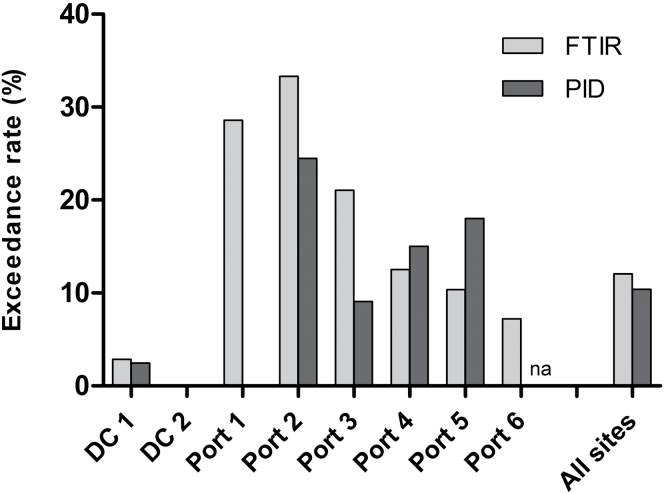
Side-by-side comparison of the ‘exceedance rate’ using PID and FTIR measurements. For the purposes of this study, the exceedance rate by PID is defined as the percentage of sampled containers with a reading > 20 p.p.m. expressed as toluene equivalents. The exceedance rate by FTIR is defined as the percentage of sampled containers with a hygienic effect above unity. The hygienic (or additive) effect is the sum of the ratio of exposure concentrations (measured value divided by 8-h OEL) of individual chemicals.

In Fig. 3, the percentage of PID readings above the arbitrarily assigned exposure limit of 20 p.p.m. is presented alongside the FTIR data from the same locations, expressed as the percentage of containers with a hygienic effect above unity. Of particular interest are the results from Port 1 where the PID method failed to identify any risk container among the 55 sampled, whereas the FTIR method identified nearly one-third as risk containers (6 of 21). The detailed analysis of the FTIR data from Port 1 showed that the risk containers had high levels of phosphine (one container), formaldehyde (two containers), carbon dioxide (three containers), and one container with both ethylene oxide and chloroethanol. Some caution must be exercised when comparing the FTIR and PID data since the sampled containers differ in number and thus, in selection.

PID and FTIR results were further compared using data from 23 containers (out of 152 analyzed) positively identified by FTIR as having levels exceeding an OEL. Only 11 (48%) of the 23 containers were also identified by PID as being >20 p.p.m. and would have been flagged as risk containers. Again, the overall analysis showed that PID failed to identify containers with hazardous levels of carbon dioxide, ethylene oxide, chloroethanol, and phosphine. Only one of the seven containers with formaldehyde was identified as hazardous by PID, and this was due to the simultaneous presence of harmless levels of 12 p.p.m. isopentane. The PID measurements successfully identified containers with high levels of terpenes and toluene. Furthermore, the PID measurements falsely identified another 7 among the 152 containers as being >20 p.p.m.; none of these had levels above any OEL according to the FTIR analyses.

**Figure 4.**
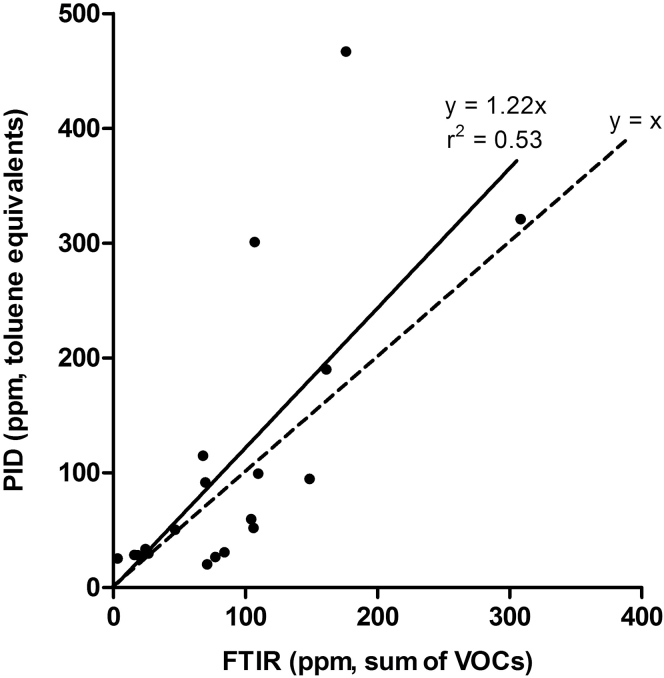
Scatter plot of PID readings > 20 p.p.m. toluene equivalents and corresponding sum of VOCs measured with FTIR spectrometry (excluding carbon monoxide, carbon dioxide, ammonia, phosphine, formaldehyde, and carbonyl sulfide). The solid line indicates the best fit by linear regression forced through origin. The broken line indicates the line of unity.

A scatter plot of PID data from all containers with PID readings > 20 p.p.m. and corresponding FTIR data (all VOCs, excluding volatile inorganic compounds) is shown in Fig. 4. Linear regression analysis indicates a poor correlation between the two methods (*r* ^2^ = 0.53, line forced through origin).

### Gas chromatography–mass spectrometry

Benzene was detected in 8 of the 21 adsorbent samples analyzed by GC–MS. One sample above the OEL was recorded at 8.5 p.p.m. (FTIR produced a similar result of 9 p.p.m.), 17 times the OEL. One sample tested at 0.3 p.p.m. benzene, whereas the remaining six samples were <0.06 p.p.m. The total VOC levels and the qualitatively identified substances largely confirmed the results from the FTIR measurements of samples from the same containers.

### Emissions from container floor

The experimental study of the off-gassing from the container floor revealed emissions of carbon monoxide (6 p.p.m.), methanol (8 p.p.m.), and formaldehyde (1 p.p.m.). This qualitative experiment confirms that these three chemicals, commonly found in container air may, at least in part, originate from natural degradation of the container floor. The accumulated concentrations in a closed container cannot be predicted from this experiment.

### Influence of sample position

**Figure 5.**
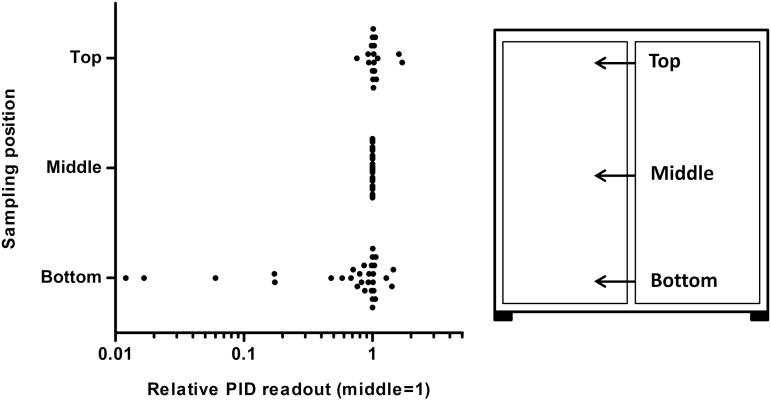
Comparison of PID readouts when sampling via the rubber seal from the same container but in different positions: bottom, middle, and top part of the door. The relative PID readout using the mid-sampling value as reference is plotted for 18 upper positions and 31 lower positions.

The VOC concentration was measured at the bottom, in the middle, and at the top of the closed container doors in 31 containers (Fig. 5). In five of these, the recorded value at the bottom was >5 times lower than in the middle level and in two of the five the recording was >50 times lower. In contrast, the middle and top samplings gave similar readings with deviations of <10%, apart from three containers where the top readings were 0.8, 1.6, and 1.7 times higher than the middle reading.

## Discussion

The wide spectrum of hazardous chemicals detected even in this limited study suggests a vast overall chemical diversity in the global fleet of containers. Hazardous off-gassing emissions were far more frequent than the rare occurrences of dedicated fumigants. The results point to significant work environment challenges where, on average, every eighth container could be considered a potential risk unit, several with carcinogens above the OELs. Noteworthy is the span of levels ranging by three to four orders of magnitude within each location. Arrival concentrations in relation to OELs provide important information for decision making, e.g. to ventilate. However, the actual personal average exposure during a work task inside a container may be substantially lower than the levels reported here and can only be established by personal monitoring (Svedberg and Johanson, 2013).

### Why so few fumigants?

The lower frequency of fumigants compared to the previous studies in Hamburg and Rotterdam (Knol-de Vos, 2002; Baur *et al.*, 2010) could partly be explained by poorer detection limits using the FTIR methodology. The principal flow of food containers, acknowledged to be frequently fumigated, was not part of the selection for this study. However, the single observation of phosphine was in fact made in a container listed as carrying rice bags, indicating that some food containers are mixed into the general flow of industrial products. The phasing out of certain fumigants, such as methyl bromide, may have had some effect on fumigation trends between the studies. Workers’ awareness and consumer demands have encouraged some importers to reduce fumigation. The development and use of alternative techniques such as ionizing radiation, heat treatment, controlled atmosphere techniques (i.e. oxygen removal by inerting with nitrogen or carbon dioxide), oxygen treatment, and the use of non-wooden pallets may have reduced the use of traditional toxic fumigants.

### Fumigants can have industrial applications

Several common industrial chemicals were detected that are also traditionally used as fumigants or disinfectants, i.e. formaldehyde, ethylene oxide, carbonyl sulfide, and carbon dioxide. However, it is also possible that these substances accumulated as a result of natural degradation processes in the goods or the emissions of residual by-products remaining from the production processes. For example, carbonyl sulfide has been used as a substitute for methyl bromide for grain fumigation (Fields and White, 2002) but also in the manufacture of, e.g., rubber (Lagzi *et al.*, 2013). Carbonyl sulfide was detected in four containers, of which two were confirmed as carrying rubber products. Paints and lacquers and products made with phenol-formaldehyde-based resins are known to emit formaldehyde (Grzeskowiak *et al.*, 1988; WHO, 1989). The experimental study confirmed that the plywood floor of the container emitted formaldehyde. The three observations of carbon dioxide above the OEL may have been residuals of inerting but may also be a result of ongoing microbiological activity.

### Location

The differences in observed levels between locations may represent geographical differences in import patterns as well as temporal randomness. The lack of extreme observations at the two distribution centers compared to the ports, as illustrated in Fig. 3, may be explained by their relatively homogeneous flow of furniture, household items, electronics, and hand tools. Both centers measured arrival concentrations in incoming containers. Any exceedance of the action levels was communicated to the manufacturers to minimize the risks in future shipments. Significant differences in air exchange rates between stationary and moving containers have been reported (Bethke *et al.*, 2013). The natural ventilation via the top corner vents during transportation from the port to the inland distribution centers may have reduced the concentration of volatiles inside the container. Random sampling in ports is probably the best strategy for establishing an understanding of the scale of the problem at the national level, while sampling immediately before the container is unpacked, or entered, is the best strategy for the protection of workers and minimizes the risk of exposure to unforeseen accumulations of off-gases.

### Sampling position

Severe underestimation of risks may result from sampling at floor level only (bottom of the doors). One plausible interpretation is that thermal convection causes fresh outside air entering at the floor level through loose or worn rubber seals to leave through the top corner vents and/or through loose or worn rubber seals at the top. This chimney effect may cause lower concentrations behind the doors, especially at the bottom, compared to sections deeper inside the container.

### Evaluation of instrumentation

Screening shipping containers for harmful substances is a considerable analytical challenge.

All methods have pros and cons, and it is important to understand their limitations in relation to the substances measured. The differences observed regarding arrival location suggest that preventive strategies, including the selection of appropriate analytical instruments, must be based on the specific conditions at each location. The results in the present and previous studies reflect random variability and true differences but also differences in detection limits, specificities, and sensitivities among analytical methods. The PID method, although quick and convenient, suffered from both false-negative and false-positive results. The shortcomings of the PID are that (i) it merely sums the chemicals present without identifying them, (ii) it has different sensitivity toward different chemicals (and zero sensitivity for some), and (iii) different chemicals have different toxic potency, reflected as different OEL values. For all these reasons, PID is less informative and may even be misleading, compared to, e.g., GC–MS or FTIR. The more accurate observations obtained by the FTIR method are explained by its better sensitivity and specificity, including the detection of low-molecular-weight compounds such as phosphine, formaldehyde, ethylene oxide, ammonia, CO_2_, and CO, all of which appeared in the present study at concentrations above the OELs. Adsorbent sampling with GC–MS analysis is a powerful method for confirmation and detailed identification of VOCs and may be useful in in-depth studies, but it is too slow for daily screening. Other methods, not used in this study, may be more suitable for convenient initial screening, e.g. multi-instruments with combinations of electrochemical detectors and PID. However, most easy-to-use handheld instruments are based on relatively non-specific detection principles. In environments with a wide spectrum of chemicals, as in container air, such instruments are likely to suffer from extensive cross-sensitivity and thus produce false positives. The likelihood of false positives increases for substances with comparatively low benchmark values, as is the case, e.g., for formaldehyde with a Swedish OEL of 0.3 p.p.m. In the present study, we learnt from the participating distribution centers that the correct identification of formaldehyde was a major challenge.

### Random observations

Some sporadic findings are worth mentioning. In agreement with previous findings (Baur *et al.*, 2010; Luyts and Mück, 2011), containers carrying shoes were over-represented among those with levels above the OELs. The frequent detection of isopentane is probably explained by its use as a blowing agent in polystyrene packing material. Isopentane was found in several units containing car batteries packed in polystyrene foam. Backtracking data from one of the distribution centers showed that boxes with ample color printing on the outside could cause accumulation of high levels of hydrocarbons.

### Long-term strategies

The best long-term approach for safeguarding workers’ health would be to eliminate harmful chemicals from production and packaging materials. Meanwhile, the high frequency of contaminated containers, the chemical complexity, and the difficult analytical challenges cannot be ignored and represent serious work safety predicaments. The results from this and previous studies clearly show the need to establish strategies for the safe handling and unpacking of shipping containers. These strategies may include not only proper sampling, analysis, and ventilation but also administrative controls such as job rotation and the use of personal protective equipment. Workers have the right to know and should be given proper information about the potential long-term risks associated with working inside shipping containers.

## Declaration

The authors declare no conflict of interest relating to the material presented in this article.

## References

[CIT0001] ACGIH (2015) TLVs and BEIs. Threshold limit values for chemical substances and physical agents & biological exposure indices. Cincinnati, OH: American Conference of Governmental Industrial Hygienists.

[CIT0002] Baur X Poschadel B Budnik LT (2010) High frequency of fumigants and other toxic gases in imported freight containers—an underestimated occupational and community health risk. Occup Environ Med; 67: 207–12. 19858536 10.1136/oem.2008.043893

[CIT0003] Bethke J Goedecke T Jahnke W (2013) Permeation through plastic dangerous goods packaging during transport in freight containers—detection of potentially explosive mixtures in containers under normal conditions of carriage. Packag Technol Sci; 26: 1–15.

[CIT0004] Fields PG White ND (2002) Alternatives to methyl bromide treatments for stored-product and quarantine insects. Annu Rev Entomol; 47: 331–59. 11729078 10.1146/annurev.ento.47.091201.145217

[CIT0005] Grzeskowiak R Jones GD Pidduck A (1988) Identification and determination of volatiles derived from phenol-formaldehyde materials. Talanta; 35: 775–82. 18964613 10.1016/0039-9140(88)80182-6

[CIT0006] Knol-de Vos T (2002) Measuring the amount of gas in import containers. Report No. 609021025/2003. Rijksinstituut voor Volksgezondheid en Milieu (RIVM) Available at http://rivm.openrepository.com/rivm/bitstream/10029/9020/1/609021025.pdf Accessed 26 September 2016.

[CIT0007] Lagzi I Mészáros R Gelybó G (2013) Astmospheric chemistry. Eötvös Loránd University Available at http://elte.prompt.hu/sites/default/files/tananyagok/AtmosphericChemistry/ Accessed 26 September 2016.

[CIT0008] Luyts L Mück O (2011) Security of containers at terminals in Benelux countries: practical experiences. Zentralbl Arbeitsmed Arbeitsschutz Ergon; 61: 408–11. Available at http://link.springer.com/article/10.1007%2FBF03345027 Accessed 26 September 2016.

[CIT0009] NIOSH (1994) Documentation for immediately dangerous to life or health concentrations. NTIS Publication No. PB-94-195047. National Institute of Occupational Health Available at http://www.cdc.gov/Niosh/idlh/intridl4.html Accessed 26 September 2016.

[CIT0010] Safe Work Australia (2012) Hazard surveillance: residual chemicals in shipping containers. Canberra, Australia: Safe Work Australia Available at http://www.safeworkaustralia.gov.au/sites/SWA/about/Publications/Documents/750/Hazard-Surveillance-Residual-Chemicals-Shipping-Containers.pdf Accessed 26 September 2016.

[CIT0011] SWEA (2015) Hygieniska gränsvärden AFS 2015:7. Stockholm, Sweden: Swedish Work Environment Authority (in Swedish).

[CIT0012] Svedberg U Johanson G (2011) Förekomst av gasformiga bekämpningsmedel och kemikalier i containrar: pilotstudie vid importkontrollen i Göteborgs hamn. Report No. 1/2011. Stockholm, Sweden: Institutet för miljömedicin, Karolinska Institutet (in Swedish). Available at http://ki.se/sites/default/files/2011-1_0.pdf Accessed 26 September 2016.

[CIT0013] Svedberg U Johanson G (2013) Work inside ocean freight containers—personal exposure to off-gassing chemicals. Ann Occup Hyg; 57: 1128–37. 23825354 10.1093/annhyg/met033 PMC3820301

[CIT0014] UNCTAD (2014) Container port throughput, annual, 2008–2014. Geneva, Switzerland: United Nations Conference on Trade and Development Available at http://unctadstat.unctad.org/wds/TableViewer/tableView.aspx?ReportId=13321. Accessed 4 December 2016.

[CIT0015] WHO (1989) Environmental Health Criteria 89 Formaldehyde. Geneva, Switzerland: World Health Organization.

